# microRNA‐27a and microRNA‐146a SNP in cerebral malaria

**DOI:** 10.1002/mgg3.529

**Published:** 2019-01-01

**Authors:** Saw Thu Wah, Hathairad Hananantachai, Jintana Patarapotikul, Jun Ohashi, Izumi Naka, Pornlada Nuchnoi

**Affiliations:** ^1^ Department of Clinical Microscopy Faculty of Medical Technology Mahidol University Bangkok Thailand; ^2^ Department of Medical Laboratory Technology University of Medical Technology Yangon Myanmar; ^3^ Faculty of Tropical Medicine Mahidol University Bangkok Thailand; ^4^ Graduate School of Science The University of Tokyo Tokyo Japan; ^5^ Center for Research and Innovation Faculty of Medical Technology Mahidol University Bangkok Thailand

**Keywords:** cerebral malaria, microRNA SNP, rs2910164, rs57095329, rs895819, Thai

## Abstract

**Background:**

During *Plasmodium falciparum* infection, microRNA expression alters in brain tissue of mice with cerebral malaria compared to noninfected controls. MicroRNA regulates gene expression post‐transcriptionally to influence biological processes. Cerebral malaria pathology caused mainly by the immunological disorder. We hypothesize that single‐nucleotide polymorphism in a microRNA influences microRNA biogenesis or target gene recognition and altering susceptibility to cerebral malaria.

**Methods:**

We performed a literature search based on immunological mechanism and applied microRNA‐related single‐nucleotide polymorphisms database to examine candidate microRNA SNPs possibly responsible for cerebral malaria. MicroRNA‐27a and microRNA‐146a are supposed to involve in cerebral malaria pathology. To assess the relationship of microRNA SNP to cerebral malaria outcome, we performed TaqMan Genotyping Assays in 110 cerebral malaria and 207 uncomplicated malaria cases for three candidate microRNA SNPs (rs895819 of microRNA‐27a, rs57095329 and rs2910164 of microRNA‐146a).

**Results:**

Our study detected no significant difference in genotype and allele frequency of individual microRNA SNPs as well as in haplotypes of microRNA‐146a between these two groups of malaria patients in Thailand. Hardy–Weinberg disequilibrium of rs57095329 in the cerebral malaria group showed a heterozygous excess which might be due to natural selection.

**Conclusion:**

Our data supported that the candidate microRNA SNPs have no major role to develop cerebral malaria.

## 1 INTRODUCTION

Cerebral malaria (CM), a life‐threatening disease caused by *Plasmodium falciparum,* is still a global health problem especially in tropical countries. The World Health Organization reported that the annual incidence of malaria was approximately 214 million and that 438,000 people died from malaria in the year 2015 (World Health Organization 2015: World malaria report 2015). Of 575,000 African children with CM, 110,000 deaths were reported (19% case fatality rate) (Murphy & Breman, 2001). The exact molecular mechanism of CM is not clearly understood. Hypotheses focus on excessive sequestration of parasitized red cells in the brain microvasculature and overproduction of inflammatory cytokines such as tissue necrotic factor (TNF) and interferon‐γ (IFN‐γ) (Prapansilp & Turner, 2013). Interestingly, the patients infected with *P. falciparum* manifest malaria of differing severities and clinical outcomes, such as uncomplicated malaria (UM), severe malaria, and CM. The scientific explanation for this remains unclear. The risk factors for disease severity are based on parasite diversity, and host age, immunity and overall health status. It is thought that human genetic factors account for 25% of the risk (Mackinnon, Mwangi, Snow, Marsh, & Williams, 2005). Evidence of genetic involvement in CM is increasingly arising from molecular analyses such as single‐nucleotide polymorphism (SNP) (Teeranaipong et al., 2008).

MicroRNA is a single‐stranded, noncoding RNA molecule, approximately 21 nucleotides in length. MicroRNA regulates gene expression at the posttranscriptional level via mRNA degradation or translational repression (Bartel, 2004). More than 60% of human protein‐coding genes are predicted to be regulated by microRNAs (Friedman, Farh, Burge, & Bartel, 2009). Single‐nucleotide polymorphisms of microRNA influence the biogenesis of microRNA and subsequently affect both the expression level of mature microRNA and target recognition. (Jazdzewski et al., 2008) During malaria disease, a differential expression of microRNA has been demonstrated in Anopheles mosquitos (Prapansilp & Turner, 2013), the brain tissue of mice with CM (El‐Assaad et al., 2011; Matin‐Alonso et al., 2018), the postmortem kidney tissue from humans (Prapansilp & Turner, 2013), and plasma from malaria patients compared to the respective nonmalarial controls (Chamnanchanunt, Fucharoen, & Umemura, 2017; Chamnanchanunt et al., 2015). These findings highlight the hypothesis that microRNA SNPs have a potential role in the severity of malaria disease (i.e., whether cerebral or not). To examine the microRNA candidate genes influencing CM, we performed a systematic literature search in the PubMed database using important keywords related to the mechanism of CM and subsequently analyzed database miRNASNP v2.0 (Gong et al., 2015) for the most probable microRNA SNPs. To the best of our knowledge, the role of microRNA SNPs in CM has not established yet. Our study will give valuable information for further genetic studies related to microRNA expression and its SNPs in the malaria patients from other ethnic populations.

## 2 METHODS

### 2.1 Subjects

A total of 317 adult patients with *P. falciparum* infection (110 CM and 207 UM) who were living in the northwest of Thailand near the border with Myanmar were examined in this study. All patients underwent treatment at the Hospital for Tropical Diseases, Faculty of Tropical Medicine, Mahidol University, Thailand. CM cases were selected from among patients with positive blood smear for asexual forms of *P. falciparum*, and suffering unarousable coma according to the criteria of the World Health Organization. The patients who had other causes of coma were excluded. Uncomplicated malaria patients were selected by positive blood smear for asexual forms of *P. falciparum* with the symptom of febrile illness without any other cause of infection. The patients who had any signs of severe malaria or evidence of vital organ dysfunction such as high parasitaemia (>100,000 parasites/μl), hypoglycemia (glucose level <22 nmol/L), severe anemia (hematocrit <20% or hemoglobin level <7 g/dl), or elevated serum creatinine (level >3.0 mg/dl) were excluded. Both UM and CM were at the age of 13 years or older. Informed consent was obtained from all patients since the first admission with *P. falciparum* malaria infection. In case of patients with age 13–16 years, the inform consent was obtained from next of kin or guardian of the participants in this study. The study was approved by the Institutional Review Board of the Faculty of Tropical Medicine, Mahidol University, Thailand.

### 2.2 SNP selection

**Figure 1:**
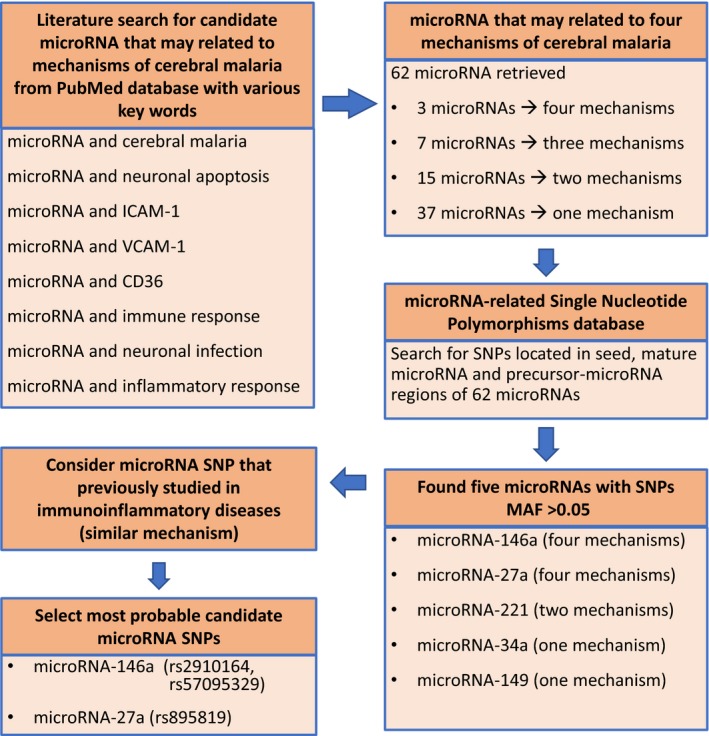
Workflow for selection of candidate microRNA SNPs

To select potential microRNA candidate genes influencing CM, a systematic literature search was conducted in the PubMed database by using various keywords: microRNA and CM, microRNA and neuronal apoptosis, microRNA and ICAM‐1, microRNA and VCAM‐1, microRNA and CD36, microRNA and immune response, microRNA and neuronal infection, and microRNA and inflammatory response. The most probable microRNA candidate genes demonstrated the involvement in nearly all of keywords. To retrieve microRNA SNPs, the existence of SNP sites in candidate microRNAs was found in the microRNA‐related single‐nucleotide polymorphisms database (miRNASNP release 2: Oct 2014) (Gong et al., 2015). The workflow for selection of candidate microRNA SNPs (miRSNP) was shown in Figure 1. The most probable microRNA with its SNPs of minor allele frequencies (MAF) >0.05 in Asian populations were selected as candidate SNPs.

### 2.3 Genotyping

Genomic DNA samples of study subjects were extracted from peripheral blood leukocytes using QIAamp Blood Kit (Qiagen, Hilden, Germany). Genotyping was performed by TaqMan SNP Genotyping Assay (Applied Biosystems, Foster City, CA, USA) according to the manufacturer's instructions. PCR amplification and genotypic discrimination were done using a LightCycler 480II (Roche Diagnostics, Mannheim, Germany). The thermal cycling and hybridization conditions for the assay were in accordance with the manufacturer's instructions. SNP calling was visualized by LightCycler 480II Endpoint Genotyping Software version 1.5.0.39 (Roche Diagnostics, Mannheim, Germany).

### 2.4 Statistical analyses

The genotypic distribution of each SNP in the two malaria groups was examined for Hardy–Weinberg Equilibrium (HWE) using a chi‐squared test. Statistical analysis was performed by using SPSS (version 18.0) for a chi‐squared test to compare the genotype and allele frequency among CM and UM patients. A *p*‐value <0.05 was considered statistically significant. The association between the genotypes and allele for the risk of CM was analyzed by calculating odds ratios (ORs) and 95% confidence interval (CI). Linkage disequilibrium (LD) and haplotype analysis were performed using Haploview software version 4.2 (Barrett, Fry, Maller, & Daly, 2005) and SNPAnalyzer 2.0 (Yoo, Lee, Kim, Rha, & Kim, 2008). Bonferroni correction test was applied to adjust the *p*‐value in case of statistically significant. To evaluate the presence of probable selection, we calculated Wright's inbreeding coefficient *F*(is) using the previously published formula *F*(is) = 1 ‐ observed heterozygosity/expected heterozygosity (Lachance, 2009).

## 3 RESULTS

### 3.1 Systematic literature search and miRSNP retrieval for selection of candidate microRNA SNPs

**Figure 2:**
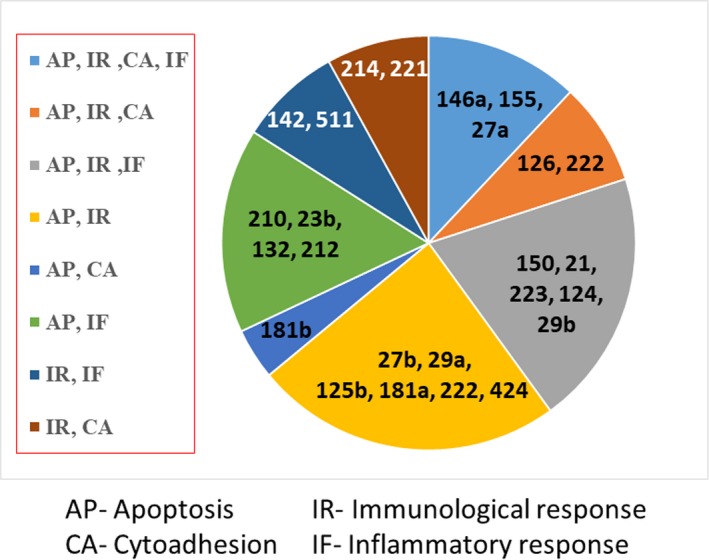
microRNAs associated with cytoadhesion, immunological response, inflammatory response, and neuronal apoptosis. The microRNA SNPs data that related to at least two of the four mechanisms are listed in the Supporting Information Table S1

From the systematic literature search, microRNAs that were mostly related to cytoadhesion, immunological response, inflammatory response, and neuronal apoptosis were shown in Figure 2 (microRNA related to two to four mechanisms). Of 62 microRNAs identified (Supporting Information Table S1 showing all microRNAs listed in the order of four to one related mechanism), microRNA‐27a, microRNA‐146a, and microRNA‐155 were found to be related to all mechanisms. Based on the microRNA SNPs database, we found five miRSNP in microRNA‐146a, microRNA‐27a, microRNA‐221, microRNA‐34a, and microRNA‐149 with minor allele frequencies (>0.05). Furthermore, we considered the previous studies of those miRSSNPs in immunoinflammatory‐related disease because of similar mechanism. Then, we selected three SNPs localized in the precursors of microRNA‐27a (rs895819) and microRNA‐146a (rs2910164), and the promoter region of microRNA‐146a (rs57095392) as the candidate miRSNPs that are potentially involved in all these critical mechanisms of CM.

**Figure 3:**
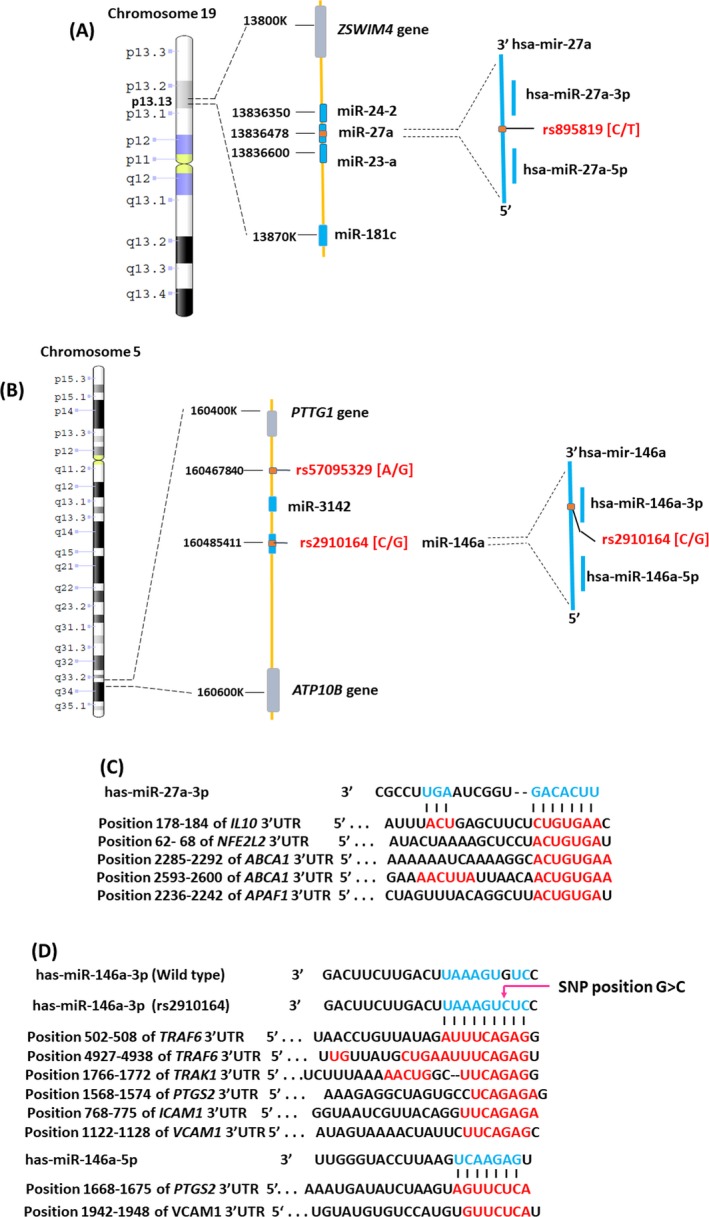
Chromosomal locations of microRNA‐27a SNP, rs895819 (a), and microRNA‐146a SNPs, rs2910164 and rs57095329 (b). Bioinformatics target gene prediction with or without experimental validation for microRNA‐27a (c) and microRNA‐146a (d). ABCA1: ATP‐binding cassette subfamily A member 1; APAF 1: apoptotic peptidase activating factor 1; ATP10B: ATPase phospholipid transporting 10B; ICAM: intercellular adhesion molecule 1; IL‐10: interleukin‐10; IRAK1: IL‐1 receptor‐associated kinase 1; NFE2L2: nuclear factor erythroid 2 like 2; PTGS2: prostaglandin‐endoperoxide synthase 2 /cyclooxygenase 2; PTTG1: pituitary tumor‐transforming 1; TRAF6: TNF receptor‐associated factor 6; UTR: untranslated region; VCAM1: vascular cell adhesion molecule 1; ZSWIM 4: zinc finger SWIM‐type containing 4

Figure 3a,b illustrates the genomic coordination of the three miRSNPs, rs895819 T>C (microRNA‐27a), rs2910164G>C (microRNA‐146a), and rs57095329 A>G (microRNA‐146a), on the chromosomes 19 and 5, respectively.

### 3.2 miRSNPs association analysis in malaria patients

**Figure 4:**
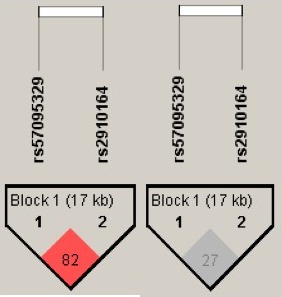
LD analysis of two microRNA SNPs displayed in D’ and *r* ^2^ by Haploview software version 4.2

To examine possible associations between candidate microRNA SNPs and CM, three candidate miRSNPs were investigated in 317 Thai patients with malaria. All patients were successfully genotyped for the three miRSNPs. The genotype distributions of the three mRSNPs in UM are in agreement with Hardy–Weinberg equilibrium (*p *>* *0.05). However, the genotype distribution of rs57095329 in the CM group showed deviation from HWE (Table 1). The distribution of genotype and allele frequencies in the CM and UM groups were compared using a chi‐squared test. No statistically significant difference was observed between the two groups (*p *>* *0.05). We did not detect any significant associations in the three miRSNPs, using various models, with the risk of CM (Table 2). LD analysis indicated that the two SNPs were in linkage equilibrium (D’ = 0.82 and *r* ^2 ^= 0.27; Figure 4). We further assessed haplotype‐based association by SNPAnalyzer 2.0 software. The estimated haplotype frequencies of rs57095329 and rs2910164 were shown in Table 3. The haplotypes of microRNA‐146a also failed to detect significant association with CM outcome after Bonferroni correction. *F*(is) values of rs57095329 and rs2910164 in the CM group were negative (−0.212 and −0.186, respectively) while in the UM group, they were positive (Table 1).

**Table 1:** Hardy–Weinberg Equilibrium (HWE) and inbreeding coefficient [*F*(is)] of miRSNPs

miRSNPs	Observed genotype			Expected genotype			HWE *p*‐value	*F*(is)
rs895819	TT	TC	CC	TT	TC	CC		
CM	47	51	12	48	49	13	0.74	−0.033
UM	104	90	13	107	83	16	0.26	−0.078
rs57095329	AA	AG	GG	AA	AG	GG		
CM	57	50	3	62	41	7	**0.035**	−0.212
UM	118	74	15	116	78	13	0.47	0.047
rs2910164	CC	CG	GG	CC	CG	GG		
CM	26	65	19	31	55	24	0.0503	−0.186
UM	66	100	41	65	102	40	0.78	0.019

Bold indicates the statistically significant difference.

**Table 2:** Genotype and allele frequencies of miRSNPs and their relation to risk of CM

Genotypes	CM *n* = 110 (%)	UM *n* = 207 (%)	*p*‐value	OR (95% CI)
rs895819 (microRNA‐27a)			0.23	
TT	47 (42.7)	104 (50.2)		
TC	51 (46.4)	90 (43.5)	0.36 (TC vs. TT)	1.25 (0.77–2.04)
CC	12 (10.9)	13 (6.3)	0.10 (CC vs. TT)	2.04 (0.87–4.81)
Dominant model	63 (57.3)	103 (49.8)	0.20 (TT vs. TC+CC)	1.35 (0.85–2.16)
Over‐dominant model	59 (53.6)	117 (56.5)	0.62 (TT+CC vs. TC)	1.12 (0.71–1.79)
Recessive model	98 (89.1)	194 (93.7)	0.15 (TC+TT vs. CC)	1.83 (0.80–4.15)
Additive model			0.1	0.74 (0.51–1.06)
Multiplicative model			0.1	1.35 (0.94–1.95)
T allele	145 (0.66)	298 (0.72)		
C allele	75 (0.34)	116 (0.28)	0.11 (C vs. T allele)	1.33 (0.93–1.89)
rs57095329 (microRNA‐146a)			0.097	
AA	57 (51.8)	118 (57.0)		
AG	50 (45.5)	74 (35.75)	0.17 (AG vs. AA)	1.40 (0.87–2.26)
GG	3 (2.7)	15 (7.25)	0.16 (GG vs. AA)	0.41 (0.11–1.49)
Dominant model	53 (48.2)	89 (43)	0.38 (AA vs. AG+GG)	1.23 (0.77–1.96)
Over‐dominant model	60 (54.5)	133 (64.25)	0.09 (AA+GG vs. AG)	1.50 (0.94–2.40)
Recessive model	107 (97.3)	192 (92.75)	0.10 (AG+AA vs. GG)	0.36 (0.10–1.26)
Additive model			0.93	0.98 (0.67–1.44)
Multiplicative model			0.93	1.02 (0.69–1.49)
A allele	164 (0.745)	310 (0.749)		
G allele	56 (0.255)	104 (0.251)	0.92 (G vs. A allele)	1.01(0.70–1.48)
rs2910164 (microRNA‐146a)			0.171	
CC	26 (23.6)	66 (31.9)		
CG	65 (59.1)	100 (48.3)	0.07(CG vs. CC)	1.65 (0.95–2.86)
GG	19 (17.3)	41 (19.8)	0.65 (GG vs. CC)	1.18 (0.58–2.39)
Dominant model	84 (76.4)	141 (68.1)	0.12 (CC vs. CG+GG)	1.51 (0.89–2.56)
Over‐dominant model	45 (40.9)	107 (51.7)	0.07 (CC+GG vs. CG)	1.55 (0.97–2.47)
Recessive model	91 (82.7)	166 (80.2)	0.58 (CG+CC vs. GG)	0.85 (0.46–1.54)
Additive model			0.48	0.89 (0.63–1.24)
Multiplicative model			0.48	1.13 (0.81–1.58)
C allele	118 (0.532)	232 (0.560)		
G allele	102 (0.468)	182 (0.440)	0.56 (G vs. C allele)	1.10 (0.79–1.53)

**Table 3:** Haplotype analysis of rs57095329 and rs2910164 for the risk of CM

Haplotype	Sequence	Frequency in CM	Frequency in UM	*p*‐value in codominant model	*p*‐value after Bonferroni correction	OR (95%CI)
H1	AC	0.5045	0.5362	0.045	0.181	0.84 (0.41–1.73)
H2	GG	0.2272	0.2270	0.542		1.001 (0.94–1.07)
H3	AG	0.2409	0.2126	0.776		1.39 (0.51–3.81)
H4	GC	0.0273	0.0242	1		0

Note

Haplotype analysis by SNPAnalyzer 2.0 version 4.2 (Yoo et al., 2008).

## 4 DISCUSSION

Despite there are many symptoms of severe malaria, CM is the most common cause of death. The pathogenesis of severe and CM is considerably difference. CM is a life‐threatening complication influencing on global health problem. In this study, we aim to investigate the role of microRNA SNP on CM. Therefore, the candidate miRNA SNPs in the present study are derived based on CM pathogenesis. In this study, the possible association of the miRSNPs in microRNA‐27a and microRNA‐146a was investigated in 110 CM and 207 uncomplicated malaria patients living in northwest of Thailand. MicroRNA is a key regulator of gene expression at the posttranscriptional gene regulation level. We hypothesized that SNPs in the microRNA genes influence microRNA biogenesis or target gene binding resulting in altered susceptibility to CM. From a literature search of the PubMed database, we found 62 microRNAs related to immunological and inflammatory responses, cytoadhesion, and neuronal apoptosis (Figure 2 and Supporting Information Table S1). Among them, three microRNAs (microRNA‐146a, microRNA‐27a, and microRNA‐155) were related to each of these mechanisms (Barker et al., 2017). Subsequent miRSNP database searching indicated that the SNP in microRNA‐155 (rs200351615 A>G) has a minor allele frequency of 0.001, too low to reliably detect in the sample size of our study. Among five microRNA SNPs with MAF >0.05 (microRNA‐146a, microRNA‐27a, microRNA‐221, microRNA‐34a, and microRNA‐149) that expected to involve in four to one mechanisms of CM, mostly are studied in various types of cancer. Finally, we chose SNPs in precursor microRNA‐27a (rs895819 T>C), precursor microRNA‐146a (rs2910164G>C) and in the promoter region of microRNA‐146a (rs57095329 A>G) as the most probable candidate gene responsible for altering susceptibility to CM.

Although gene frequencies in the control group conformed with HWE (*p *>* *0.05), in the CM group, rs57095329 deviated from HWE (*p *=* *0.035) and rs290164 was borderline in deviation (*p *=* *0.0503). Deviation of the HWE test may be based on the presence of population stratification (Cardon & Palmer, 2003), genotyping error (Hosking et al., 2004), natural selection (Lachance, 2009), or be a sign of disease association (Lee, 2003). However, the HWE test cannot distinguish among these causes of deviation. Population stratification was not the cause, as these samples showed genetic homogeneity in HLA typing (Hananantachai et al., 2005). In addition, if a genotyping error existed in a locus, the HWE deviation would have increased with increasing sample size (Sen & Burmeister, 2008). Our results showed increased deviation of HWE in the study group despite its smaller sample size compared to that of the control group. So, genotyping error was unlikely to be the reason for deviation from HWE. The statistical genetics of Wright's inbreeding coefficient *F*(is) test can detect selection‐induced departures from HWE (Lachance, 2009). The considerable negative *F*(is) values in the study group of rs57095329 (−0.212) and rs2910164 (−0.186) indicated the excess of heterozygosity, while the positive *F*(is) values in the control group suggest deficits of heterozygosity. The negative correlation between *F*(is) among cases and controls is a sign of selection (Sen & Burmeister, 2008). Our data suggest homozygous fitness in the control group and heterozygous disadvantage in the study group of rs57095329 that was likely selected by previous malaria disease in the Thai population. However, further studies are needed to confirm this finding in other populations residing in malaria‐endemic areas.

The linkage disequilibrium analysis among the two SNPs of microRNA‐146a showed low LD values (*r* ^2^ = 0.27). Thus, the two markers are independently related to each other. We found no significant differences in the genotype and allele frequencies of the three individual candidate genes between malaria groups. The haplotypes of the two SNPs in microRNA‐146a also showed no association with malaria manifestation.

No one has reported investigations of the effect of microRNA‐27a polymorphism in CM. In our study, no significant differences were observed in genotype and allele frequencies in various models. MicroRNA‐27a is intergenic and clusters with microRNA‐23a and microRNA‐24‐2 on the short arm of chromosome 19 (19p13.13: 13836440‐13836517). Its SNP, rs895819, is located on the terminal loop of the precursor microRNA‐27a (Figure 3a). Han et al. demonstrated that the variation in the terminal loop of microRNA slightly impairs DiGeorge Syndrome Critical Region 8 (DGCR8) binding during microRNA biogenesis (20). However, the mutant type is able to compete with wild type for primary‐microRNA processing and might not be affected on the level of mature microRNA expression (Han et al., 2006). So, rs895819 might not effect on the expression of microRNA‐27a and this may explain the absence of an association with risk of CM.

The findings of the literature and bioinformatics analyses indicate that microRNA‐27a has a putative binding site for *IL‐10*,* NFE2L2/Nrf2, ABCA1,* and *APAF1*, and negatively regulates target gene expression (Figure 3c). Xie et al. demonstrated that higher expression of microRNA‐27a enhances the inflammatory response of macrophages by downregulating the anti‐inflammatory cytokine *IL‐10* and upregulating proinflammatory cytokines *IL‐1*β*, IL‐6, IL‐12,* and *TNF* (Xie et al., 2014). In addition, microRNA‐27a regulates *Nrf2* in neuronal, SH‐SY5Y cell and NRF2 systems are important in the regulation of neuroinflammation in response to oxidative stress in the brain (Narasimhan et al., 2012). Moreover, *Nrf2* promotes CD36 expression on macrophages (Olagnier et al., 2011) and decreases expression of CD36 associated with CM (Cserti‐Gazdewich et al., 2012). The increased expression of microRNA‐27a might lead to a greater risk of CM by interacting with *IL‐10, Nrf2*. This evidence is consistent with increased expression of microRNA‐27a in brain tissue of the CM mice model (El‐Assaad et al., 2011). In contrast, upregulation of microRNA‐27a inhibits *ABCA1* (Zhang et al., 2014) and *APAF1* genes (Chen et al., 2014) that may protect against CM by inhibiting microparticle formation and apoptosis mechanisms. Since *ABCA1* involves the process of microparticle formation and *ABCA1* gene knockout mice show complete protection from CM by reduced levels of circulating TNF and microparticles. (Combes et al., 2005) MicroRNA‐27a negatively regulates the *APAF1* gene at the posttranscriptional level and inhibits neuronal apoptosis (Chen et al., 2014).

In regard to microRNA‐146a, bioinformatics target gene prediction and experimental validation indicated that it has a putative binding site on the 3′UTR of *TRAF6, IRAK1, PTGS2/COX2, VCAM1,* and *ICAM1* (Figure 3d). MicroRNA‐146a negatively regulates the nuclear factor NF‐κB signaling pathway by inhibiting the expression of *TRAK1* and *TRAF6* (Wu et al., 2015). NF‐κB downstream effects include gene regulation of several different mechanisms such as cytoadhesion, immuno‐inflammatory responses, and neuronal apoptosis. Wu et al., (2015) demonstrated that increased microRNA‐146a in human cerebral microvascular endothelial cells decreases expression of VCAM1 and subsequently reduces T‐cell adhesion by inhibiting NF‐κB. Human astroglial cells transfected with microRNA‐146a mimic downregulate the mRNA of *COX2, TNF, IL‐6*, and *IL‐8* (Li et al., 2011). COX‐2 expression is significantly increased in brain endothelial cells and astrocytes of patients who died with CM compared to that in brain parenchyma of controls (Deininger, Kremsner, Meyermann, & Schluesener, 2000). In addition, the mean plasma TNF level was the higher in fatal cases of CM compared to both surviving cases of CM and cases of uncomplicated malaria in Gambian children (Kwiatkowski et al., 1990). Also, El Gazzar, Church, Liu, & McCall, (2011) reported that microRNA‐146a regulates both transcriptional silencing and translational disruption of TNF in the THP‐1 sepsis cell model. Lu et al., (2010) demonstrated that microRNA‐146a is highly expressed in Treg cells and negatively regulates immune cell function by targeting *Signal transducer and activator transcription 1* (*Stat1*), a key molecule in the IFN‐γ signaling pathway. Moreover, microRNA‐146a downregulation is associated with neuron apoptosis by the elevation of the protein levels of IRAK1 and TRAF6 in peripheral neuropathy and cultured dorsal root ganglia neurons in the mouse model of type 2 diabetes (Wang et al., 2014). These findings suggest that microRNA‐146a acts as an anti‐inflammatory cytokine and that its upregulation might give protection from CM.

MicroRNA‐146a is intergenic between the *PTTG1* and *ATP10B* genes, and situated on the long arm of chromosome 5 (5q34: 160485352‐160485450) (Figure 3b). The two functional SNPs in microRNA‐146a, rs2910164 and rs57095329, influence the expression levels of mature microRNA (Jazdzewski et al., 2008; Luo et al., 2011). It is well known that microRNA‐146a is a regulator of immune and inflammation responses, and its functional polymorphism has been studied in several immuno‐inflammatory diseases such as multiple sclerosis (MS) (Li et al., 2015), systemic lupus erythematosus (SLE) (Fu et al., 2016), rheumatoid arthritis (RA) (Fu et al., 2016), and Alzheimer's disease (Cui et al., 2014). However, no one has reported on the relationship between microRNA‐146a SNPs and CM. We observed no significant association of microRNA‐146a polymorphisms with CM outcome in Thai population. The expression of microRNA‐146a varies in the different alleles of rs2910164. The expression of microRNA‐146a increases with the C allele of rs2910164 in patients with relapsing‐remitting MS (RMMS) (Li et al., 2015). In contrast, Jazdzewski K et al. reported that this C allele of rs2910164 causes decreased expression of microRNA‐146a compare to the G allele by mispairing within the hairpin and decreasing stability due to a lowering of free energy. Interestingly, the GC heterozygous state produces more mature microRNAs, one from the leading strand (microRNA‐146a), two from the passenger strand (microRNA‐1461*G and microRNA‐1461*C), compared to the CC and GG homozygous states. Since the SNP is located in the seed region of the passenger strand, both isoforms in the CG genotype might determine the expression of potential targeted genes (Jazdzewski et al., 2009). Although more efficient inhibition of the targeted genes might occur in heterozygous than homozygous states, our results show a higher risk of CM in the heterozygous state.

According to the report of similar association studies in immuno‐inflammatory diseases, rs2910164 and rs5095329 show inconsistent results in association of the disease risk and level of microRNA‐146a expression (Cui et al., 2014; Fu et al., 2016; Li et al., 2015; Luo et al., 2011). In MS, no association is observed with rs57095329 although the AA genotype shows increased expression of microRNA‐146a. In contrast, rs2910164 in RRMS is significantly associated with risk of disease in female patients. Interestingly, C alleles of rs2910164 in RRMS confer higher expression of microRNA‐146a and increased plasma levels of TNF and IFN‐γ (Li et al., 2015). This finding is inconsistent with the El Gazzar et al., (2011) and Lu et al., (2010) reports that microRNA‐146a is a negative regulator of inflammatory and immune response. Meta‐analysis indicated that rs2910164 is not significantly associated with the risk of SLE and RA (Fu et al., 2016). However, rs57095329 is significantly associated with SLE (Fu et al., 2016). Similarly, the AA genotype of rs57095329 is associated with cognitive decline in Alzheimer patients while there is no significant association observed with rs2910164 (Cui et al., 2014). The above results show inconsistence in the association of two functional SNPs with the risk of immuno‐inflammatory diseases despite altering the expression of microRNA‐146a in different genotypes. This discrepancy may be due to the incomplete understanding of the consequences of polymorphism on mature microRNA expression and on inflammatory responses. This might be a reason for the lack of a significant association between microRNA‐146a SNP and CM. On the other hand, the polymorphism might have no causal role in the expression of microRNA‐146a but rather might associate with a nearby causal variant since the other function gene of microRNA‐146a, rs2431697. It is situated between the *PTTG1* and microRNA‐146a, and the risk allele is correlated with lower expression of microRNA in European SLE patients (Lofgren et al., 2012). These inconsistency evidences between populations highlighted the necessary of functional analysis in each specific ethnic population.

To sum up, microRNA‐27a and microRNA‐146a are related to the mechanisms of immuno‐inflammatory response, cellular adhesion, and neuronal apoptosis. So, we expected that SNPs related to those microRNAs may be associated with CM. However, the candidate microRNA SNP shows no association with CM in a Thai population. In addition, another text‐mining‐derived microRNAs (miR‐221, miR‐341and miR‐149) should be investigated in future study in order to complete the scenario of systematic study. Our studies are limited in the determination of microRNA‐27a and microRNA‐146a expression levels in the malaria patients. Thus, to know the effects of microRNA polymorphism in CM, further study is warranted on microRNA expression together with the corresponding functional SNPs in differing ethnic groups. The genotype–phenotype relationship is crucial and should be evaluated in functional study. More specifically, it is needed to elucidate the functional relevance of microRNA SNP on mature microRNA expression. Although, we selected the most probable microRNA SNPs that related to CM, the rest of microRNA SNP (MAF >0.05) are also interested to be examined. Moreover, polymorphism in microRNA‐146a‐targeted genes (*IRAK1* and *TRAF6*) in the NF‐κB pathway should be considered for further association studies of CM.

## CONFLICT OF INTEREST

The authors declare that we have no conflicts of interest.

## AUTHOR CONTRIBUTION

STW: performed the experiments, analyzed the data, wrote the paper, and prepared figures and/or tables. HH: performed the experiments and analyzed the data. JP: conceived and designed the experiments. IN: performed the experiments and analyzed the data. JO: performed the experiments and analyzed the data. PN: conceived and designed the experiments, performed the experiments, analyzed the data, wrote the paper, prepared figures and/or tables, proofread and finalized drafts of the paper.
