# Supplementary material for: microRNA‐27a and microRNA‐146a SNP in cerebral malaria
Source: Mol Genet Genomic Med. 2019 Jan 1;7(2):e00529. doi: 10.1002/mgg3.529 (PMC6393659; doi:10.1002/mgg3.529)
Supplement: Supplementary file 1 [file MGG3-7-na-s001.docx]

**Supplementary Table S1. miRNAs SNP in various regions with minor allele frequency**

| No. | miR Name | SNP in seed region | SNP in mature miRNA except seed | SNP in pre-miR except mature miRNA | Allele | MAF |
| --- | --- | --- | --- | --- | --- | --- |
| 1 | 27a |  |  | rs895819 | T/C | 0.314(HCB) |
|  |  |  |  |  |  | 0.371(JPT) |
|  |  |  |  | rs11671784 | G/A | 0.012 |
| 2 | 146a |  |  | rs2910164 | G/C | 0.465(HCB) |
|  |  |  |  |  |  | 0.389(JPT) |
| 3 | 155 | rs200351615 |  |  | A/G | 0.001 |
|  |  |  |  | rs140021681 | T/C | 0.0001 |
| 4 | 126 |  | rs199992070 |  | C/T | 0.001 |
| 5 | 222 |  |  | rs72631825 | G/A | 0.013 |
|  |  |  |  | rs187612279 | G/A | 0.0008 |
|  |  |  |  | rs191727254 | T/A,C | 0.0001 |
| 6 | 223 |  |  | rs186354597 | G/A | 0.004 |
|  |  |  |  | rs34952329 |  | No Data |
| 7 | 124-2 |  | rs72631829 |  | C/T | 0.005 |
| 8 | 29b |  | rs200396959 |  | C/T | 0.001 |
| 9 | 27b |  |  | rs192552111 | C/T | 0.002 |
| 10 | 125b |  |  | rs200232194 | G/T | 0.001 |
| 11 | 221 | rs113054794 |  |  | A/C | 0.5 |
| 12 | 23b |  |  | rs201848546 | G/A | 0.008 |
| 13 | 181b |  |  | rs200492369 | A/T | 0.001 |
| 14 | 29a | No | No | No |  |  |
| 15 | 150 | No | No | No |  |  |
| 16 | 21 | No | No | No |  |  |
| 17 | 181a | No | No | No |  |  |
| 18 | 210 | No | No | No |  |  |
| 19 | 212 | No | No | No |  |  |
| 20 | 214 | No | No | No |  |  |
| 21 | 132 | No | No | No |  |  |
| 22 | 142 | No | No | No |  |  |
| 23 | 424 | No | No | No |  |  |
| 24 | 511 | No | No | No |  |  |
| 25 | Let-7a |  | rs201607747 |  | T/A | 0.001 |
| 26 | 16-1 |  |  | rs72631826 | T/C | 0.001 |
| 27 | 15b | rs192595529 |  |  | G/A | 0.001 |
|  |  |  |  | rs146020563 | A/G | 0.001 |
| 28 | 34a |  | rs35301225 |  | C/T | 0.5 |
|  |  |  |  | rs201359809 | C/G | 0.001 |
|  |  |  |  | rs72631823 | G/A | 0.005 |
| 29 | 107 |  |  | rs199975460 | T/C | 0.001 |
| 30 | 125a |  | rs143525573 |  | G/A | 0.00001 |
| 31 | 145 | rs190323149 |  |  |  | No data |
| 32 | 147b |  |  | rs56073218 | C/G | 0.001 |
| 33 | 148b |  |  | rs185315720 | C/T | 0.001 |
|  |  |  | rs74878365 |  |  | No data |

**Supplementary Table S1. miRNAs SNP in various regions with minor allele frequency (cont.)**

| No | miR Name | SNP in_seed region | SNP in mature miRNA except seed | SNP in pre-miR except mature miRNA | Allele | MAF |
| --- | --- | --- | --- | --- | --- | --- |
| 34 | 149 |  |  | rs71428439 | A/G | 0.175 |
|  |  |  |  | rs2292832 | T/C | 0.279(HCB) |
|  |  |  |  |  |  | 0.372(JPT) |
| 35 | 181c |  |  | rs201394123 | G/A | 0.001 |
| 36 | 199a-1 | rs183664554 |  |  | A/G | 0.001 |
|  | 199a-2 |  |  | rs147011290 | C/T | 0.001 |
| 37 | 200b |  |  | rs72563729 | G/A | 0.016 |
| 38 | 339 |  | rs72631820 |  | A/G | 0.005 |
|  |  |  | rs145196722 |  | C/T | 0.0001 |
|  |  |  |  | rs13232101 |  | No data |
|  |  |  |  | rs72631831 | G/A | 0.01 |
| 39 | 495 |  |  | rs149411304 | C/T | 0.002 |
|  |  |  |  | rs144777170 | A/G | 0 |
|  |  |  |  | rs201464875 | C/T | 0.001 |
|  |  |  | rs199783139 |  | C/T | 0.001 |
| 40 | 505 | rs143213653 |  |  | C/T | 0.001 |
|  |  |  |  | rs202116296 | C/A,G,T | 0.0008 |
| 41 | 17~92 (18a) |  |  | rs41275866 | C/T | 0.00001 |
|  |  |  |  | rs201226336 | A/G | 0.001 |
|  | 17~92 (20a) |  |  | rs185831554 | G/T | 0.0008 |
|  | 17~92(92b-1) |  | rs9589207 |  | A/G | 0 |
|  |  |  | rs72631821 |  | C/T | 0.005 |
| 42 | Let-7b | No | No | No |  |  |
| 43 | Let-7c-5p | No | No | No |  |  |
| 44 | Let-7i | No | No | No |  |  |
| 45 | 15a | No | No | No |  |  |
| 46 | 17-3p | No | No | No |  |  |
| 47 | 19b | No | No | No |  |  |
| 48 | 22 | No | No | No |  |  |
| 49 | 23a | No | No | No |  |  |
| 50 | 29c | No | No | No |  |  |
| 51 | 31 | No | No | No |  |  |
| 52 | 128a | No | No | No |  |  |
| 53 | 134 | No | No | No |  |  |
| 54 | 139-5p | No | No | No |  |  |
| 55 | 200c | No | No | No |  |  |
| 56 | 203 | No | No | No |  |  |
| 57 | 206 | No | No | No |  |  |
| 58 | 343 | No | No | No |  |  |
| 59 | 347 | No | No | No |  |  |
| 60 | 374 | No | No | No |  |  |
| 61 | 451 | No | No | No |  |  |
| 62 | 497 | No | No | No |  |  |
